# Exploring the Sleep-Sarcopenia Relationships: Results from the Longitudinal Study of Ageing and Health in Viet Nam

**DOI:** 10.1093/geroni/igaf122.3097

**Published:** 2025-12-31

**Authors:** Thai Le, Tuo Yu Chen, Cong Nguyen Vu, Yasuhiko Saito

**Affiliations:** Taipei Medical University, Taipei City, Taiwan (Republic of China); Academia Sinica, Taipei City, Taiwan (Republic of China); Institute of Population, Health and Development, Ha Noi, Ha Noi, Vietnam; Nihon University, Tokyo, Tokyo, Japan

## Abstract

Existing research demonstrates conflicting findings on the relationship between nighttime sleep (sleep quality and quantity) and sarcopenia. Meanwhile, the effects of daytime napping on sarcopenia remain unclear. This study utilized data from the Longitudinal Study of Ageing and Health in Viet Nam to investigate the relationships between sleep with sarcopenia. This cross-sectional study comprised 2,863 adults aged 60 + (mean-age 68.84±7.08 years, 54.7% female). Sarcopenia was assessed using the criteria of the Asian Working Group for Sarcopenia (AWGS) 2019. Self-reported sleep measures included sleep duration (short sleep [< 6 hours], normal sleep [6-9hours], long sleep [>9hours]), sleep satisfaction (yes/no), non-restorative sleep (yes/no), total number of insomnia complaints (scored 1-3, higher score indicating severe insomnia), and daytime napping (none, short nap [< 1hour], long nap [>1hour]). Logistic regression analysis was used to analyze the data, adjusting for sociodemographic and health covariates. A total of 549 participants (19.3%) were diagnosed with sarcopenia, with greater prevalence in males. Short sleep (OR = 1.23,95%CI=1.00-1.50), long sleep (OR = 2.29,95%CI=1.30-4.03), and total number of insomnia complaints (OR = 1.09,95%CI=1.00-1.19) were significantly related to an increased likelihood of sarcopenia. Sleep satisfaction (OR = 0.81,95%CI=0.66-0.99) and daytime napping >1 hour (OR = 0.79,95%CI=0.63-0.99) were related to reduced risks of sarcopenia risk. Non-restorative sleep was not related to sarcopenia. When combining all sleep parameters in the analysis, only long sleep was significantly related to increased risks of sarcopenia (OR = 2.41,95%CI=1.37-4.25). Long sleep was found to raise the risks of sarcopenia among older adults. More research using data from different cohorts is needed to investigate the longitudinal relationships between sleep measures and sarcopenia.

